# Daily Stressors and Memory Lapses: Considering Positive Affect as a Stress Buffer

**DOI:** 10.1093/geroni/igaf122.493

**Published:** 2025-12-31

**Authors:** Zeynep Saruhanlioglu, Yvonne Brehmer, Keri Pekaar, Dorien Kooij, Jacqueline Mogle, David Almeida, Patrick Klaiber

**Affiliations:** Tilburg University, Tilburg, Noord-Brabant, Netherlands; Tilburg University, Tilburg, Noord-Brabant, Netherlands; Tilburg University, Tilburg, Noord-Brabant, Netherlands; Tilburg University, Tilburg, Noord-Brabant, Netherlands; RTI Health Solutions, Durham, North Carolina, United States; The Pennsylvania State University, University Park, Pennsylvania, United States; Tilburg University, Tilburg, Noord-Brabant, Netherlands

## Abstract

Memory lapses are common in adulthood and can negatively affect daily functioning. Stressor occurrence has been previously linked to same-day memory lapses, yet prior research has not distinguished between retrospective memory (RM) and prospective memory (PM) lapses or examined whether positive affect buffers these effects. RM primarily depends on retrieving stored information, whereas PM requires not only retrieval but also monitoring and execution processes, that may be more susceptible to stressors. This study investigated whether positive affect moderates the within-person relationship between daily stressor occurrence and retrospective and prospective memory lapse occurrence among middle-aged and older adults. Data were drawn from 1,125 community-dwelling adults (ages 43-90; M = 62.43) in the National Study of Daily Experiences 3. Participants completed eight consecutive daily telephone interviews assessing stressor occurrence, retrospective and prospective memory lapses, and positive affect. Results from mixed-effects logistic regression models indicated that experiencing a memory lapse was significantly more likely on stressor days than on stressor-free days. However, this association was only observed for retrospective memory lapses, not prospective ones. While positive affect did not buffer the daily stressor-memory lapse link, individuals with higher overall positive affect reported fewer memory lapses across both memory types. The findings highlight that memory retrieval processes may be more vulnerable to stressor-related disruptions, whereas individuals may employ compensatory strategies to maintain daily PM functioning. Furthermore, they underscore the need to explore modifiable factors to protect against daily memory lapses throughout adulthood.

